# Red Seaweed (*Gracilaria verrucosa* Greville) Based Polyurethane as Adsorptive Membrane for Ammonia Removal in Water

**DOI:** 10.3390/polym14081572

**Published:** 2022-04-12

**Authors:** Salfauqi Nurman, Saiful, Rahmi, Binawati Ginting, Marlina

**Affiliations:** 1 Graduate School of Mathematics and Applied Sciences, Universitas Syiah Kuala, Banda Aceh 23111, Indonesia; salfauqi@mhs.unsyiah.ac.id; 2 Department of Agricultural Industrial Engineering, Faculty of Agricultural Technology, Universitas Serambi Mekkah, Banda Aceh 23245, Indonesia; 3 Department of Ship Engineering, Politeknik Pelayaran Malahayati, Aceh Besar 23381, Indonesia; 4 Department of Chemistry, Faculty of Mathematics and Natural Sciences, Universitas Syiah Kuala, Banda Aceh 23111, Indonesia; rahmi@fmipa.unsyiah.ac.id (R.); binawati@unsyiah.ac.id (B.G.); marlina@unsyiah.ac.id (M.); 5 Research Center for Environmetal and Natural Resources, Universitas Syiah Kuala, Banda Aceh 23111, Indonesia

**Keywords:** red seaweed, polyurethane membrane, adsorption, chemisorption, ammonia

## Abstract

Polyurethane membranes are widely developed polymers by researchers because they can be made from synthetic materials or natural materials. Red seaweed (*Gracilaria verrucosa* Greville) is a natural material that can be developed as a raw material for polyurethane membranes. This study used red seaweed biomass (RSB) as a raw material to manufacture polyurethane as an adsorptive membrane for removing ammonia in water. The membrane composition was determined using the Box–Behnken design from Response Surface Methodology with three factors and three levels. In the ammonia adsorption process, the adsorption isotherm was determined by varying the concentration, while the adsorption kinetics was determined by varying the contact time. Red seaweed biomass-based polyurethane membrane (PUM-RSB) can adsorb ammonia in water with an adsorption capacity of 0.233 mg/g and an adsorption efficiency of 16.2%. The adsorption efficiency followed the quadratic model in the Box–Behnken design, which resulted in the optimal composition of RSB 0.15 g, TDI 3.0 g, and glycerin 0.4 g with predicted and actual adsorption capacities of 0.224 mg/g and 0.226 mg/g. The ammonia adsorption isotherm using PUM-RSB follows the Freundlich isotherm, with a high correlation coefficient (R^2^) of 0.977, while the Langmuir isotherm has a low R^2^ value of 0.926. The Freundlich isotherm indicates that ammonia is adsorbed on the surface of the adsorbent as multilayer adsorption. In addition, based on the analysis of adsorption kinetics, the adsorption phenomenon follows pseudo-order II with a chemisorption mechanism, and it is assumed that the bond that occurs is between the anion –SO_4_^2−^ with the NH_4_^+^ cation to form ammonium sulfate (NH_4_)_2_SO_4_ and between isocyanates (NCO) with NH_4_^+^ cations to form substituted urea.

## 1. Introduction

Researchers are increasingly developing and exploring polymers to meet human needs. The polyurethane polymer is an especially promising and common research subject. Polyurethane is a type of polymer formed in the presence of an intermediate reaction when a monomer containing an isocyanate group (–N=C=O) is combined with another monomer containing a hydroxyl group (–OH); the reaction that occurs is an addition reaction that forms a urethane bond (–NHCOO–) [1,2]. Polyurethane continues to be developed from various raw materials such as synthetic materials [3] and natural materials [4]. In addition, polyurethane applications are also being developed such as foams, elastomers, films, coatings, membranes, and others.

One such innovation is a polyurethane membrane. Polyurethane membranes have many advantages, including minimal energy usage, not requiring additional chemical substances [5], and strong chemical resistance [6], making it viable in extreme pH conditions; additionally, the membrane is very easy to apply, practical, and straightforward [3]. The use of natural materials as raw materials for the manufacture of polyurethane membranes has been widely developed, such as the use of castor oil [7] and carrageenan [8], as well as many other materials that have been developed for the synthesis of polyurethane membranes. Previous studies have reported results on the synthesis of polyurethane membranes from various types of base materials used for various applications, such as a research report from Huang and Lai in 1995, which stated that membranes derived from hydroxy-terminated polybutadiene (HTPB) and 4,4’-diphenylmethane diisocyanate (MDI) and 2,4-toluene diisocyanate (TDI) with various polyols had been used for oxygen gas separation. The membrane synthesized using MDI and 1,4,-butanediol resulted in a hydrogen-bonding index (HBI) value of 2.257 and a separation factor of 2.46 cm^2^/s cmHg [9].

Polyurethane membranes have also been synthesized by reacting oxidized fatty acids and free fatty acids from castor oil with a 2,4-toluene diisocyanate (TDI), treated or oxidized without protection. The resulting optimum condition is that the ratio of methylated-oxidized free fatty acids and 2,4-toluene diisocyanate is 1.2:0.56 (mol/mol), with a polymerization temperature of 100 °C for 5 min. This produces membranes with transparent, homogeneous, elastic, and strong properties, with a glass transition point of 127.4 °C and tensile strength of 41.7 MPa [7]. In addition, a polyurethane membrane was created from carrageenan with 2,4-toluene diisocyanate (TDI) with a composition of 15% carrageenan from TDI and a polymerization temperature of 60 °C for 5 min. The polyurethane membrane produced has an elongation percentage of 9%, a tensile strength of 340 kgf/mm^2^, a glass transition temperature of 243.6 °C, and a melting point of 423.02 °C. The polyurethane membrane has elastic, transparent, and strong properties, with a rejection factor of 45.9% and a flux value of 39.2 L/h.m^2^. The membrane was applied to the ultrafiltration process of 1000 ppm dextran solution [8]. Polyurethane membranes from natural materials have better performance when compared to membranes from commercial polyurethane materials. This is based on comparing two polyurethane membranes synthesized from castor oil and 4-4-dicyclohexyl methane diisocyanate and commercial polyurethane thermoplastics [3].

Apart from being applied as a filter, the polyurethane membrane can also be applied as an adsorbent or as an adsorptive membrane; the study results show that polyurethane foam is modified with chitosan and can be used as an adsorbent in mercury-contaminated well water from Aceh Jaya District. Hg (II) adsorption using polyurethane foam matched the Freundlich isotherm model with an R-value of (0.9417). Although the adsorption constant (Kf) was low at 0.297 mg/g, the polyurethane foam adsorbent was able to adsorb Hg from well water samples up to 83.1% [10]. Recent studies have shown that the membrane can be used as an adsorptive membrane to adsorb ammonia in wastewater, through hollow fiber ceramic membranes based on natural zeolite through phase inversion and sintering techniques, removing 90% of ammonia in wastewater with a flux of 249.57 L/m^2^·h and mechanical strength of 50.92 Mpa [11]. The research was continued by looking at the effect of the sintering temperature and the pH of the solution. The behavior of the adsorption kinetics showed that the adsorption process was most suitable for the second-order kinetic model with an adsorption capacity of 12.468 mg/g. The membrane’s morphology and negatively charged surface have contributed significantly to high ammonia adsorption (96.7%). The maximum adsorption activity was achieved at pH 7 with a 0.4 g/L membrane in 120 min [12]. Polyurethane membrane as an adsorptive membrane has been synthesized from algae biomass. The membrane can absorb ammonia in pond water. The addition of activated carbon can increase the functional groups as well as the surface area, which is important for NH_3_–N removal. The adsorption capacity increased rapidly after activating carbon was added to the polyurethane membrane (from 187.84 to 393.43 µg/g). In addition, this study shows that activated carbon can be used as a filler in AlgPU. The use of algae developed for polyurethane membranes has demonstrated the potential to remove NH_3_–N [13]. Polyurethane membrane applications have also been developed to reduce ammonia levels from various sources and may reducing ammonia levels in the air [14], river water [15], wastewater [16], saline wastewater [17], and NH_4_Cl solutions [13], as well as from an ammonia gas sensor [18].

The use of red seaweed (*Gracilaria verrucosa* Greville) biomass as raw material for the synthesis of polyurethane membranes in this study cannot be separated from the content of the hydroxyl group (–OH) as a urethane bond-forming (–NHCOO–) [19,20], which is widely contained in carrageenan, alginate, and agar of said biomass [21,22,23]. Seaweed is one of the export commodities that has great potential. Types of seaweed that have high economic value include red algae (*Rhodophyceae*) and brown algae (*Phaeophyceae*). Rhodophyceae is a type of seaweed that produces carrageenan and agar, while Phaeophyceae is a type of seaweed that produces alginate [22]. *Gracilaria verrucosa* Greville is a species of *Rhodophyceae* of the genus *Gracilaria*, which can produce agar and carrageenan. Carrageenan that can be produced reaches 60–75% depending on the growing location and the extraction method used [21]. In addition, red seaweed biomass is used for the synthesis of polyurethane as an ammonia adsorptive membrane, because red seaweed biomass contains carrageenan, which has the anion –SO_4_^2–^, which can be utilized to bind NH_4_^+^ cations in water to form ammonium sulfate [24,25]. The composition of the material greatly influences the formation of the hard and soft segments of the polyurethane membrane [26,27]. Determination of the composition of the polyurethane membrane synthesis uses Response Surface Methodology (RSM) with the Box–Behnken Design. The design uses three factors and three levels. The three factors used are red seaweed biomass (RSB), toluene-2,4-diisocyanate (TDI), and glycerin. RSB and TDI are the essential ingredients for forming urethane bonds, while glycerin is used as a plasticizer and crosslinker [28].

## 2. Materials and Methods

### 2.1. Instruments and Materials

The instruments used in this study were a UV–Vis Spectrophotometer (UV-1700 pharmaspec, SHIMADZU, Tokyo, Japan) and SEM (JEOL-6510 LA, Tokyo, Japan). The materials used were 1,4-dioxane (99.8%) and distilled water as a solvent, glycerin (98%), and castor oil as a plasticizer. Toluene-2,4-diisocyanate (TDI) (80%, remainder 2,6-diisocyanate) was used as a reagent in the synthesis of polyurethane. NH_4_Cl was used to prepare the model ammonia solution. All the chemicals were obtained from Merck (Darmstadt, Germany). Red seaweed (*Gracilaria verrucosa* Greville) biomass (RSB) was obtained from fish ponds in the Lamnga area, Mesjid Raya District, Aceh Besar District, Aceh Province, Indonesia.

### 2.2. Treatment Design

The treatment design in this study was the same as the treatment design of our previous study, which had differences in treatment responses [19,20]. Synthesis of red seaweed biomass-based polyurethane membrane (PUM-RSB) was conducted using Box–Behnken Design from Response Surface Methodology on Software Design Expert Version 10.0.3.0. This design uses three factors (RSB, TDI, and glycerin), three levels (low, medium, and high), and two responses (adsorption capacity and adsorption efficiency), as shown in Table 1. Box–Behnken Design’s combination design produced 17 runs, as shown in Table 2.

### 2.3. Polyurethane Membrane Fabrication

The fabrication of polyurethane membranes also followed the procedure of our previous study [19,20]. RSB was weighed according to the combination design table, put into a beaker, combined with 5 g of 1,4-dioxane and 0.5 g of castor oil, then stirred until homogeneous for 10 min; then TDI and glycerin were added according to the combination design. The polymerization process was carried out at 60 °C for 2 h while stirring. The resulting dope solution was poured into a closed petri dish and placed in a dust-free room at room temperature for 24 h. After the membrane sheet was formed, the membrane was released from the mold with the help of warm distilled water for 1–2 h.

### 2.4. Adsorption Process

The test solution used was NH_4_Cl based on SNI. 06-6989.30-2005 (2005) [29]. NH_4_Cl was dried at 100 °C for 2 h, and then 3.819 g was weighed and put into a 1000 mL flask diluted with distilled water to obtain 1000 ppm ammonia mother liquor. Then 10 mL of the mother liquor was pipetted and diluted using distilled water in a 1000 mL flask to obtain a test solution of 10 ppm. A 70 mL test solution was taken and put into 17,100 mL glasses, and each glass was inserted with PUM-RSB based on the experimental design with a size of 3 cm × 3 cm (0.3577 g). The adsorption process was carried out at room temperature, neutral pH (7–8), and stirred for 60 min. The Nessler method was used to analyze ammonia levels before and after the adsorption process with a wavelength of 425 nm.

### 2.5. Adsorption Capacity and Efficiency

The adsorption capacity and efficiency can be calculated by measuring the concentration of the ammonia solution before and after the adsorption process, using Equations (1) and (2).

Adsorption capacity:

(1) $$\mathrm{Qe}=\left(\frac{\mathrm{Co}-\mathrm{Ca}}{m}\right)\times V$$

Adsorption efficiency:

(2) $$E=\left(\frac{\mathrm{Co}-\mathrm{Ca}}{\mathrm{Co}}\right)\times 100\%$$

where:

Qe: Adsorption capacity (mg/g)

Co: Initial concentration of adsorbate (ppm)

Ca: Final concentration of adsorbate (ppm)

V: Volume of solution (L)

m: Weight of adsorbent (g)

E: Adsorption efficiency (%)

### 2.6. Adsorption Isotherm

Adsorption isotherms, including Langmuir and Freundlich isotherms, can produce the maximum capacity of the adsorption process caused by a single layer (monolayer) of the adsorbate on the surface of the adsorbent. Langmuir and Freundlich isotherms can be determined using Equations (3) and (4).

Isotherms Langmuir:

(3) $$\mathrm{Qe}=\frac{\mathrm{Qm}\;\mathrm{Kl}\;\mathrm{Ca}}{1+\mathrm{Kl}\;\mathrm{Ca}}$$

Isotherms Freundlich:

(4) $$\mathrm{Qe}={\mathrm{KfCa}}^{1/n}$$

Description

Ca: Adsorbate concentration (ppm)

Qe: Adsorption capacity (mg/g)

Kl: Langmuir’s constant (L/mg)

Qm: Maximum capacity (mg/g)

Kf: Freundlich’s constant (mg/g)

1/n: Adsorption intensity

### 2.7. Adsorption Kinetics

The adsorption kinetics can be obtained by determining the changes in the adsorbate concentration, and the value of k (slope) is plotted on the graph. The adsorption speed will affect the adsorption kinetics and can be defined as the amount of substance that is adsorbed per unit time [30]. The reaction rate equations for the adsorption system in pseudo-order I and pseudo-order II kinetics are shown in Equations (5) and (6), respectively.

Pseudo-order I:

(5) $$\log\;\left(\mathrm{Qe}-\mathrm{Qt}\right)=\log\mathrm{Qe}-\frac{\mathrm{Kt}}{2.303}t$$

Pseudo-order II:

(6) $$\frac{t}{\mathrm{Qt}}=\frac{1}{{\mathrm{KQe}}^{2}}+\frac{t}{\mathrm{Qe}}$$

Description

Qe: Adsorption capacity (mg/g)

Qt: Adsorption capacity at time t (mg/g)

K: Adsorption rate constant (g/mg.min)

t: Adsorption time (minutes)

## 3. Results and Discussion

### 3.1. Ammonia Adsorption

**Figure 1:**
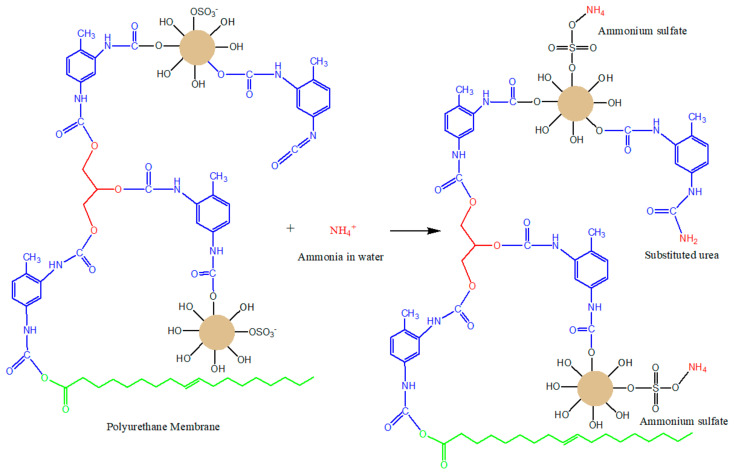
Schematic illustration reaction of ammonia in water by PUM-RSB forms ammonium sulfate and substituted urea.

The decrease in ammonia levels in water from the adsorption process using PUM-RSB can be seen in Table 2. Ammonia levels that could be adsorbed by PUM-RSB were minimal, with the highest adsorption capacity of 0.233 mg/g and adsorption efficiency of 16.2% PUM-RSB. PUM-RSB was applied for the adsorption process in sheet form; the adsorbent in sheet form has a small surface area when compared to the adsorbent in powder form, resulting in a small adsorption capacity of the polyurethane membrane. [31,32]. The ammonia adsorption process on polyurethane membranes follows the binding reaction of NH_4_^+^ cations by SO_4_^2−^ anions from carrageenan in seaweed to ammonium sulfate and binding of NH_4_^+^ cations by isocyanate groups (–NCO) of excess toluene diisocyanate to substituted urea [24,25]. The reaction that occurs can be seen in Figure 1.

### 3.2. Statistical Design Model

This study used a quadratic design model because it has a higher R^2^ value for adsorption capacity response than the 2FI, linear, and cubic models. The value of R^2^ expressed in percentage can show the contribution of the regression; a relatively good R^2^ value is above 70%. The greater the value of R^2^, the more significant the contribution of factor (x) to the response (y) [33]. Although the cubic model has a high R^2^ value, it does not have a PRESS value and a Pred R^2^ value, so that the effect of each variable that has a different signal is not different or aliased [34]. The statistical design model of adsorption capacity can be seen in Table 3, with the analysis of variance seen in Table 4.

**Figure 2:**
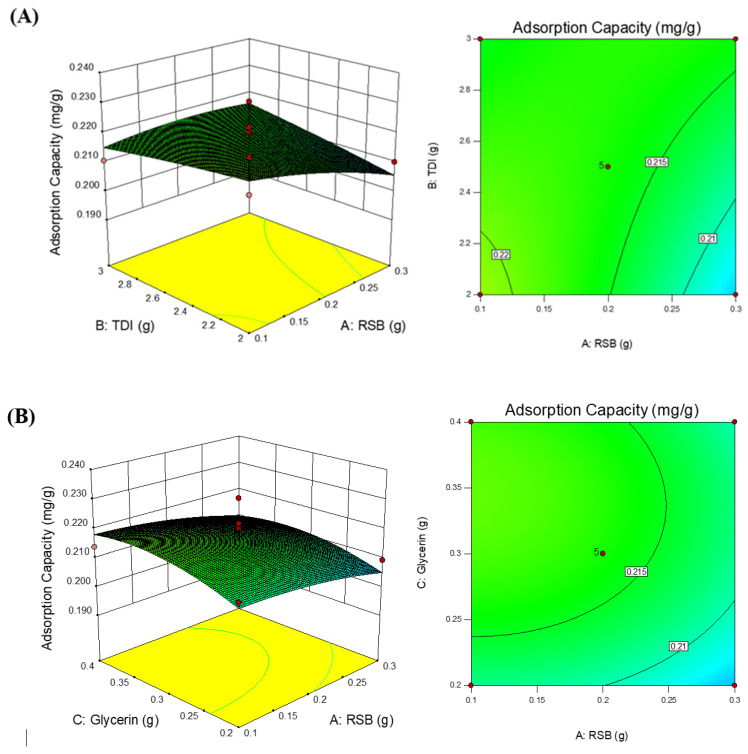

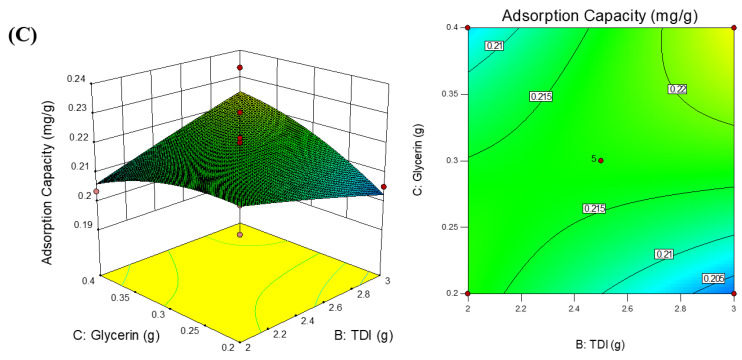
A 3D plot and surface plot of the relationship between (**A**) RSB and TDI on adsorption capacity, (**B**) RSB and glycerin on adsorption capacity, and (**C**) TDI and glycerin on adsorption capacity.

**Figure 3:**
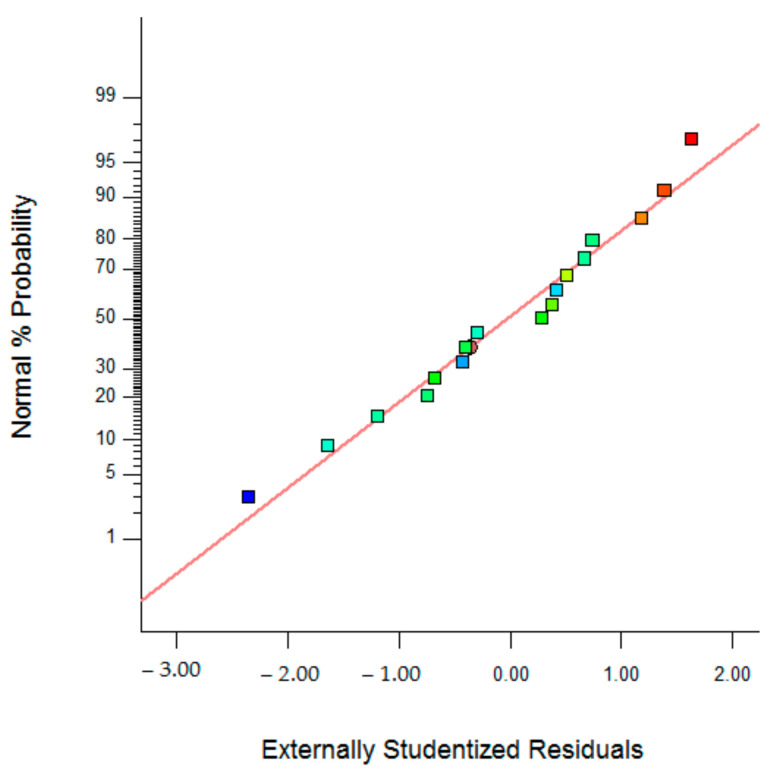
The residual normal plot of the optimum design of the PUM-RSB synthesis from Box–Behnken Design.

The Pred R-Squared value of 0.3894 and Adj R-Squared value of −0.3956 had differences above 0.18; the expected difference was less than 0.2. The Adeq Precision value is the ratio of signal to noise. The expected ratio was greater than 4; in all models, the resulting ratio was less than 4; this indicated an inadequate signal, so that all of these models were not able to navigate the design space [35]. The relationship between adsorption capacity with factor (x) 3D plot and the normal plot of residue can be seen in Figure 2 and Figure 3.

### 3.3. Polyurethane Membrane Optimization

**Figure 4:**
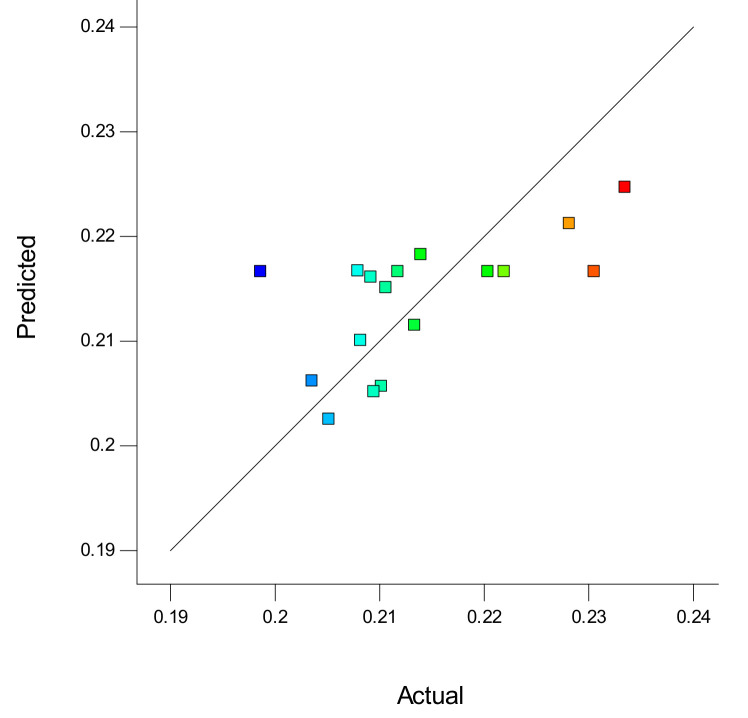
Predictive and actual graphs of the optimum design of the PUM-RSB synthesis from Box–Behnken Design.

The optimization results using Box–Behnken Design from Response Surface Methodology provided a PUM-RSB composition solution, as shown in Table 5 and Figure 4. The table also shows the predictive and actual adsorption capacity using PUM-RSB, which were 0.224 mg/g and 0.226 mg/g; The predicted and actual results did not have a significant difference, and it shows that the optimal composition obtained from Box–Behnken Design could be used for the synthesis of polyurethane membranes. The optimum solution given had a desirability value of 0.820; this value was close to the expected value of 1 [36]. The results of another study showed that polyurethane from AlgPU had an adsorption capacity of 187.84 µg/g and increased to 393.43 µg/g after the addition of 6% (*w*/*w*) activated carbon [13]. The addition of activated carbon can be increased the adsorption capacity of PUM-RSB.

**Figure 5:**
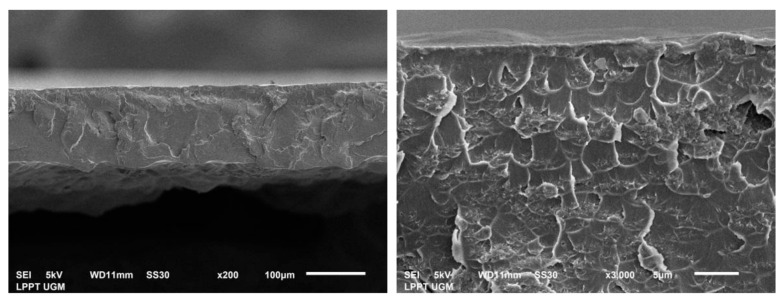
Cross-section of PUM-RSB with 200 and 3000× magnification.

The optimal morphology analysis of PUM-RSB was determined through cross section using a scanning electron microscope (SEM). As seem om the SEM results in Figure 5, the polyurethane membrane had a tight structure on the surface, making it difficult to penetrate the test solution into the membrane. This membrane structure caused more adsorption on the membrane surface [27,37,38].

### 3.4. Ammonia Adsorption Using Optimal Polyurethane Membrane

**Figure 6:**
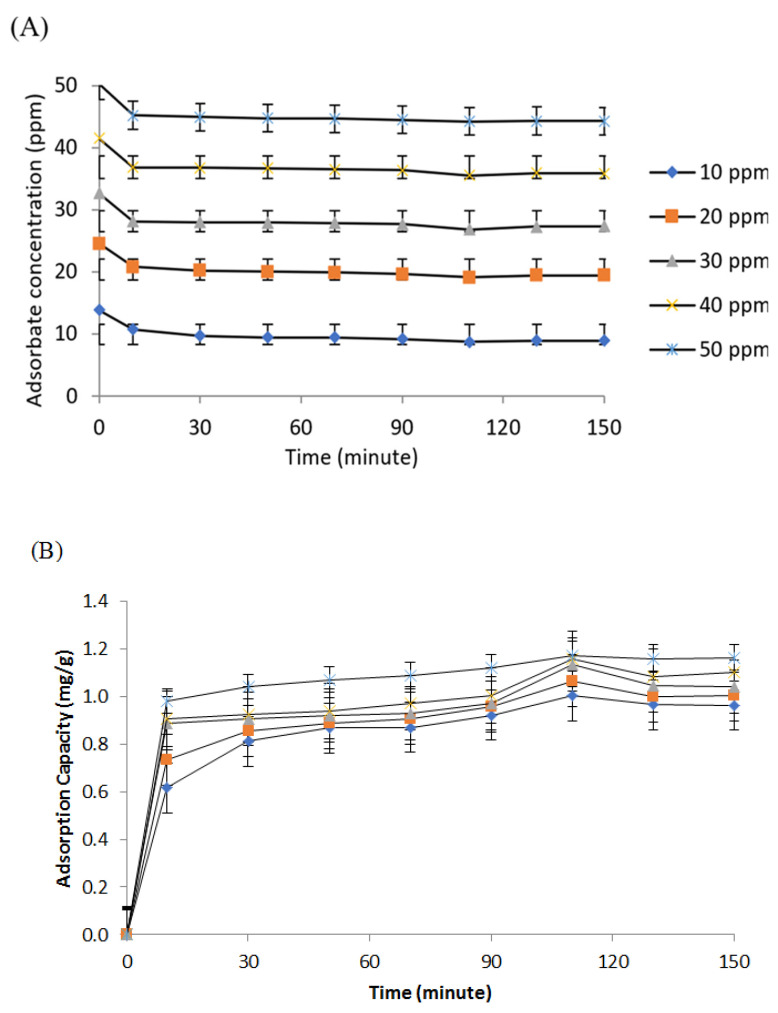

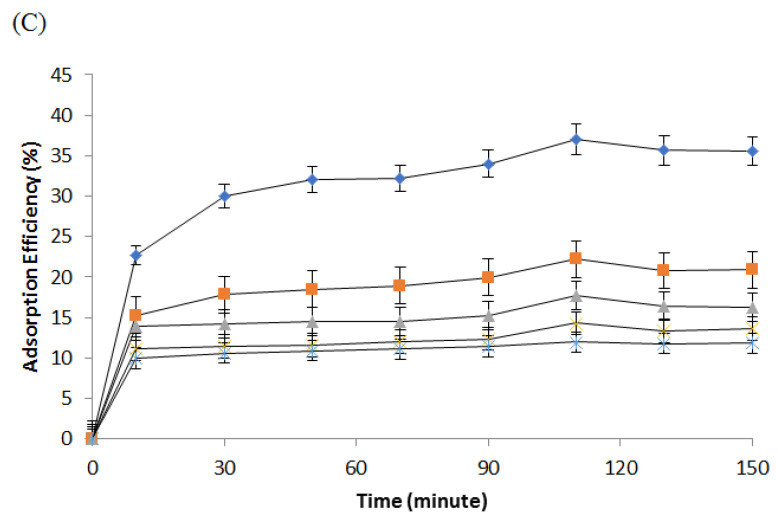
Graph of decrease in adsorbate concentration (**A**), adsorption capacity (**B**), and polyurethane membrane adsorption efficiency (**C**).

The optimal PUM-RSB produced was then used for the adsorption process of ammonia in water with variations in ammonia concentration and contact time. The NH_4_Cl solution with five concentrations was used, namely 10, 20, 30, 40, and 50 ppm. The adsorption process was carried out with time variations of 10, 30, 50, 70, 90, 110, 130, and 150 min. Time variation is carried out based on the principle that the longer the contact time, the higher the amount of adsorbate will be adsorbed [39]. The adsorption capacity of ammonia in the first 10 min for all concentrations increased rapidly, as seen in Figure 6B. This initial adsorption phase was primarily regulated by the diffusion of adsorbate carried by water to the adsorbent surface [10,40]. All the adsorbate concentrations used showed the maximum value trend for adsorption capacity and efficiency at a contact time of 110 min. The decrease in adsorption capacity and efficiency values was observed at 130 min and was thought to be due to the influence of the stirring process during adsorption on the multilayer adsorbate. Multilayer adsorption has weak bonds in the upper layer, so that the adsorbate on the outer layer after reaching equilibrium can be rereleased due to the influence of the stirring process; as a result, the efficiency and adsorption capacity decreased after reaching the maximum point.

### 3.5. Adsorption Isotherm

Adsorption isotherm is a curve that can describe the phenomenon of regulation of retention (release) or mobility of a substance from an aqueous medium to the solid phase at constant pH and temperature [41]. The adsorption equilibrium, which is the ratio between the amount adsorbed and the rest in the solution, can be formed when the adsorbent phase has been in contact with the adsorbent for a sufficient time so that it will establish a dynamic equilibrium between the concentration in the liquid phase and the interface concentration in the solid phase [39]. The Langmuir isotherm adsorption equation was used as an empirical model assuming monolayer adsorption and a non-linear equation. Therefore the adsorption process can only occur in a limited number of identical and equivalent locations where the steric resistance between the adsorbed molecules is in adjacent parts [42]. In its derivation, the Langmuir isotherm refers to homogeneous adsorption in which each molecule has a constant enthalpy and the same activation energy of adsorption, without any transmigration of the adsorbate on the surface [39].

**Figure 7:**
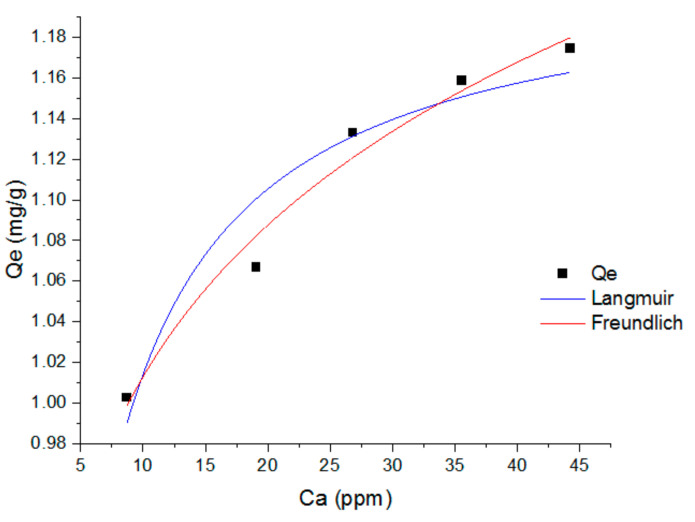
Langmuir and Freundlich isotherm graph for ammonia adsorption using PUMP-RSB at contact time of 110 min.

The graph of the Langmuir isotherm that used a non-linear form at the optimum contact time (110 min) for ammonia adsorption using PUM-RSB can be seen in Figure 7 and Table 6. The Langmuir isotherm had a correlation coefficient (R^2^) of 0.926. A low contact time caused the adsorption capacity (Qm) not to reach the maximum value for adsorption, while at a higher contact time, the adsorption capacity could reach a higher value because the contact time between the adsorbate and adsorbent was longer [25] for the Qm and Kl values from the non-linear Langmuir equation, namely 1.21478 ± 0.02203 and 0.505 ± 0.09597, respectively.

The Freundlich isotherm is the initial form of the relationship known to describe non-ideal and reversible adsorption upon multilayer formulation [39,41]. In the Freundlich isotherm, the amount adsorbed is the sum of the adsorption in all parts, with the stronger binding site occupied first, until the adsorption process is complete, and the adsorption energy will decrease exponentially [25]. The Freundlich isotherm analysis of ammonia adsorption using PUM-RSB can be seen in Figure 7 and Table 6. These results show that the adsorption process occurred in multilayer because it had an R^2^ value greater than the Langmuir equation, which was 0.977. The slope between 0 and 1 measures the adsorption intensity or surface heterogeneity, becoming more heterogeneous as the value approaches zero. Moreover, a value close to 1 implies a chemisorption process and above 1 indicates cooperative adsorption [39]. The Kf and n resulting from the Freundlich equation were 0.79976 ± 0.02405 and 9.74569 ± 0.88071, respectively.

### 3.6. Adsorption Kinetics

Adsorption is the process of attaching adsorbate molecules to the surface of the adsorbent. The curve that can describe the rate of retention or release of adsorbate from the aqueous environment to the solid phase interface at a certain dose, temperature, flow rate, and pH of the adsorbent is referred to as adsorption kinetics. There are two main processes involved during adsorption: physical (physisorption) or chemical (chemisorption). Physical adsorption occurs due to weak attractive forces (van der Waals), whereas chemisorption involves the formation of bonds or reactions between the adsorbate and the adsorbent involving electron transfer. In addition, chemical adsorption is irreversible, so that it only forms a single layer (monolayer) [43,44].

The adsorption kinetics equation includes pseudo-reaction order, rate constant, and activation energy. The pseudo-order of the reaction was determined by using linear equations of pseudo-order I and pseudo-order II of the graph plotting between the (x) axis as adsorption time (t) and the (y) axis as adsorption capacity, namely Log (Qe − Qt) for pseudo-order I and Log Qe for pseudo-order II. Thus, that will obtain a linear equation: y = ax + b, where the slope is the value of the adsorption rate constant. In addition, a linear regression value (R^2^) was obtained, which indicated the suitability of the data with the kinetic model.

**Figure 8:**
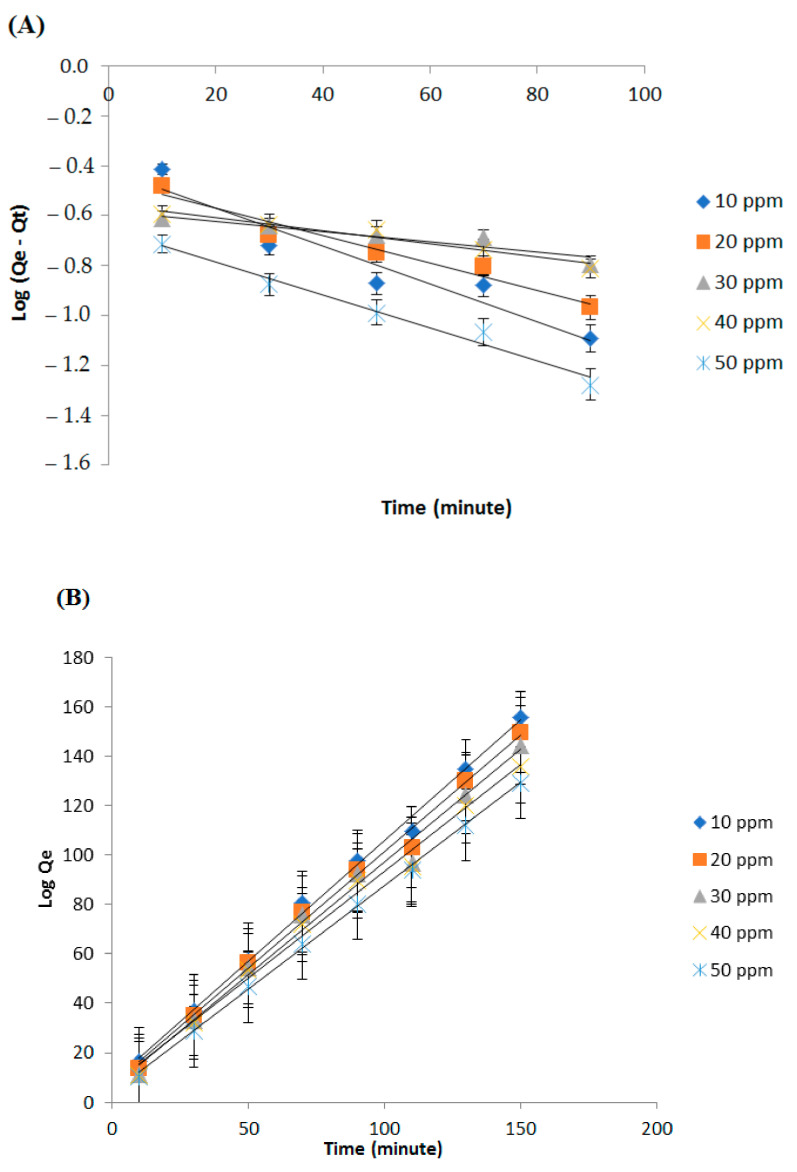
Graph of pseudo-order I (**A**) and pseudo-order II (**B**) adsorption kinetics at each concentration.

The results of the adsorption kinetics analysis using pseudo-order I and pseudo-order II can be seen in Figure 8 and Table 7. From these results, it can be seen that the adsorption process followed a pseudo-order II model. This is based on the higher R^2^ value. In the pseudo-order II equation, the mechanism of adsorption is chemisorption, namely the formation of chemical bonds between the adsorbate and the adsorbent. In this study, it was assumed that the adsorption process occurred chemically, namely by the formation of bonds between the –SO_4_^2−^ anion and NH_4_^+^ cations to form ammonium sulfate (NH_4_)_2_SO_4_ and between isocyanate (NCO) and NH_4_^+^ cations to form substituted urea (Figure 1) [24,45].

## 4. Conclusions

Red seaweed of the *Gracilaria verrucosa* Greville species is one of the natural materials that have the potential to be used as raw material for the synthesis of polyurethane membranes. The polyurethane membrane composition can be determined using the Box–Behnken Design from Response Surface Methodology with the quadratic model. The PUM-RSB can be applied as an adsorbent in the adsorption process of ammonia in water. The adsorption process of ammonia in water using a polyurethane membrane resulted in an adsorption capacity of 0.226 mg/g. The ammonia adsorption process using PUM-RSB followed the Freundlich isotherm and multilayer adsorption. Moreover, based on the analysis of adsorption kinetics, the adsorption phenomenon followed pseudo-order II with a chemisorption mechanism. The PUM-RSB membrane can be an alternative type of adsorbent for removing ammonia in water.

## Figures and Tables

**Table 1:** PUM-RSB synthesis design levels with 3 factors (x) and 3 levels with Box–Behnken Design.

Factor	Parameter	Levels		
		Low (–)	Medium (0)	Height (+)
x_1_	RSB (g)	0.1	0.2	0.3
x_2_	TDI (g)	2.0	2.5	3.0
x_3_	Glycerin (g)	0.2	0.3	0.4

RSB: red seaweed biomass; TDI: toluene-2,4-diisocyanate.

**Table 2:** A decrease in ammonia levels from the adsorption process in response to adsorption capacity and efficiency.

Run	Factor 1 A: RSB (g)	Factor 2 B: TDI (g)	Factor 3 C: Glycerin (g)	Response Adsorption Capacity (mg/g)	Response Adsorption Efficiency (%)
1	0.2	3.0	0.4	0.233	16.2
2	0.2	2.5	0.3	0.222	11.8
3	0.3	2.5	0.4	0.208	6.0
4	0.2	2.0	0.2	0.208	5.9
5	0.2	3.0	0.2	0.205	4.6
6	0.1	2.5	0.4	0.214	8.5
7	0.2	2.5	0.3	0.212	7.6
8	0.1	2.0	0.3	0.228	14.2
9	0.3	2.0	0.3	0.210	6.9
10	0.1	2.5	0.2	0.213	8.3
11	0.3	2.5	0.2	0.209	6.6
12	0.2	2.5	0.3	0.199	1.5
13	0.2	2.0	0.4	0.204	3.9
14	0.3	3.0	0.3	0.209	6.5
15	0.2	2.5	0.3	0.220	11.2
16	0.2	2.5	0.3	0.231	15.1
17	0.1	3.0	0.3	0.211	7.1

**Table 3:** The statistical value of the adsorption capacity design model.

Source	2FI	Linear	Quadratic	Cubic
Std. Dev	0.010	0.010	0.012	0.012
R-Square	0.340	0.120	0.389	0.618
Adj R-Square	−0.056	−0.084	−0.396	−0.527
Pred R-Square	−0.760	−0.451	−3.257	N/A
Adeq Precision	3.444	2.653	2.506	2.838
PRESS	2.58 × 10^−3^	2.21 × 10^−3^	6.49 × 10^−3^	N/A

**Table 4:** ANOVA analysis for quadratic model of adsorption capacity.

Source	Sum of Squares	df	Mean Square	F Value	*p*-Value Prob > F	Characterization
Model	5.937 × 10^−4^	9	6.597 × 10^−5^	0.50	0.838	Not significant
A-RSB	1.054 × 10^−4^	1	1.054 × 10^−4^	0.79	0.403	
B-TDI	9.344 × 10^−6^	1	9.344 × 10^−6^	0.07	0.799	
C-Glycerin	6.764 × 10^−5^	1	6.764 × 10^−5^	0.51	0.499	
AB	6.841 × 10^−5^	1	6.841 × 10^−5^	0.51	0.496	
AC	8.625 × 10^−7^	1	8.625 × 10^−7^	6.486 × 10^−3^	0.938	
BC	2.671 × 10^−4^	1	2.671 × 10^−4^	2.01	0.199	
A^2^	1.213 × 10^−5^	1	1.213 × 10^−5^	0.09	0.771	
B^2^	7.310 × 10^−7^	1	7.310 × 10^−7^	5.497 × 10^−3^	0.943	
C^2^	5.760 × 10^−5^	1	5.760 × 10^−5^	0.43	0.532	
Residual	9.308 × 10^−4^	7	1.330 × 10^−4^			
Lack of Fit	3.488 × 10^−4^	3	1.163 × 10^−4^	0.80	0.556	not significant
Pure Error	5.820 × 10^−4^	4	1.455 × 10^−4^			
Cor Total	1.525 × 10^−3^	16				

**Table 5:** PUM-RSB synthesis optimum composition solution from Box–Behnken Design.

RSB (g)	TDI (g)	Glycerin (g)	Adsorption Capacity (Predicted) (mg/g)	Desirability	Adsorption Capacity (Actual) (mg/g)
0.15	3.0	0.4	0.224	0.820	0.226

**Table 6:** Langmuir and Freundlich isotherm data for ammonia adsorption using PUMP-RSB at a contact time of 110 min.

Isotherm Models	Parameters	Value
Langmuir	Adjusted-R-Square	0.902
	R-Square (COD)	0.926
	Reduced Chi-Sqr	5.01 × 10^−4^
	Kl	0.505 ± 0.09597
	Qm	1.21478 ± 0.02203
Freundlich	Adjusted-R-Square	0.970
	R-Square (COD)	0.977
	Reduced Chi-Sqr	1.54 × 10^−4^
	Kf	0.79976 ± 0.02405
	n	9.74569 ± 0.88071

**Table 7:** Rate constant data and kinetics of pseudo-order I and pseudo-order II ammonia adsorption using PUM-RSB.

Kinetics Models	Parameters	Concentration (ppm)				
		10	20	30	40	50
Pseudo order I	Adjusted-R^2^	0.951	0.978	0.787	0.929	0.970
	K (g/mg.min)	0.016	0.012	0.014	0.011	0.020
	Qe (mg/g)	0.640	0.620	0.664	0.630	0.565
Pseudo order II	Adjusted-R^2^	0.997	0.994	0.989	0.990	0.999
	K (g/mg.min)	0.118	0.129	0.1378	0.109	0.192
	Qe (mg/g)	1.025	1.060	1.096	1.155	1.194

## Data Availability

Not applicable.
